# Eating Behavior and Caries Experience in Children with Growth Stunting

**DOI:** 10.1055/s-0042-1758069

**Published:** 2022-12-13

**Authors:** Regina Puspita Sari, Laili Rahayuwati, Arlette Suzy Setiawan

**Affiliations:** 1 Faculty of Dentistry, Universitas Padjadjaran, Jatinangor, Indonesia; 2 Department of Community Nursing, Faculty of Nursing, Universitas Padjadjaran, Jatinangor, Indonesia; 3 Department of Pediatric Dentistry, Faculty of Dentistry, Universitas Padjadjaran, Bandung, Indonesia

**Keywords:** caries experience, eating behavior, preschool, stunting

## Abstract

**Objective** Stunting is the impaired growth and development children experience from chronic malnutrition and repeated infection that cause long-term damage. Malnutrition for a long time will affect the shape and composition of bones and teeth, making children more vulnerable to dental health problems. Preschoolers with sufficiently active consumption can choose the food they like delightful foods so that it can increase the risk of caries. Based on data from the Bandung City Health Office in 2019, 161 toddlers (25.43%) in Sukawarna Village experienced stunting. This study analyzed the relationship between eating behavior and caries experience in stunting children in Sukawarna Village, Sukajadi District.

**Materials and Methods** This study uses a quantitative descriptive research method with a secondary data analysis approach from the documentation of the Universitas Padjadjaran Academic Leadership Grant data with the title “Aspects of jaw growth-development and family approach in early detection and prevention of stunting.” Sampling used purposive sampling specifically for preschool children with a sample size of 80 respondents. The data obtained will be processed and then analyzed using the Spearman Rank correlation statistical test.

**Results** Sixty-three percent of respondents have poor eating behavior, and the majority (80.35%) have cavities due to caries (decay). Spearman Rank correlation coefficient is −0.145 and significance is 0.0983.

**Conclusion** Overall eating behavior is related to the caries experience in stunting children. Consumption of cariogenic foods influences the caries experience.

## Introduction

Malnutrition is a common problem in some developing countries and includes being underweight, stunting, wasting, and micronutrient deficiencies. 1 Growth stunting is caused by chronic malnutrition and recurrent disease that causes long-lasting damage leading to children growing shorter than the standard height for their age. Prolonged nutrition deficiency will cause irreversible physical and cognitive abnormality, metabolic disorders that increase the risk of children experiencing obesity and diabetes, and dental health problems. 2 3 Growth stunting starts in the first 1,000 days of a child's life, intrauterine until approximately 2 years old. Indonesia is included in the second-highest prevalence of stunting in the Southeast Asia Region after Cambodia. The prevalence of stunting in Indonesia in 2018 was 30.8%, then in 2021 it was reported that the prevalence of stunting was 24.4%. Although it is decreasing, the figure is still high because the World Health Organization (WHO) target is 20%. 4 5 6

The preschool age period (3–5 years) is the initial period for developing children's abilities. Children will learn about behavior, eating habits, and food during this period. 7 According to the Minister of Health of the Republic of Indonesia No. 41 of 2014, eating behavior is an essential aspect of life as it can affect long-term health because the quality and quantity of food and drinks consumed will affect nutritional status. The nutritional status of children under 5 is an integral part of health indicators. If there is a nutritional problem in this golden age, it is difficult to return it even though the following nutritional needs are met. 8 Inadequate nutritional status in children will affect the shape and composition of bones and teeth. Chronic undernutrition has been associated with disturbed dental development, including enamel hypoplasia and delayed eruption, so children are more susceptible to demineralization and caries. 9 10 Preschoolers, as sufficiently active consumers, can choose the food, they like, especially sweet foods so that it can increase the risk of caries. 2

Dental health is one of the most important things because it is the first entry point for food. Caries is a dental health problem that often occurs in children. Caries occurs because food residue is attached to the teeth, which causes cavities. 11 Indonesian health research data in 2018 showed that the prevalence of dental and oral problems in Indonesia has increased compared with 2013, from 25.9% in 2013 to 57.6% in 2018. The prevalence of dental and oral problems in Bandung City is 50.02%. 12 13 In children who experience growth stunting, there is a decrease in the salivary flow because of the atrophy of salivary glands; it affects self-cleaning abilities and increases the risk of caries. 9 10 Dental health, nutritional intake, and eating behavior are closely related to the growth and development phase in children. A balanced nutritional intake plays a role in supporting dental health, and dental health plays a vital role in adequate nutritional intake. 14 15 16 Based on the Decree of the Mayor of Bandung in 2020, Sukawarna Village is the village with the fourth-highest risk of stunting in Bandung City. 17 Therefore, this study aimed to analyze the relationship between eating behavior and caries experience in stunting children in Sukawarna Village, Sukajadi District.

## Materials and Methods

### Study Design

A quantitative descriptive study with a secondary data analysis approach was conducted from September 2020 to March 2021. The secondary data were collected from the modified WHO food frequency questionnaire and Decayed, Missing, Filled Teeth Index (DMFT) scores from the documentation of the Universitas Padjadjaran Academic Leadership Grant data.

### Eligibility Criteria

The population of this study was 164 data of children aged under 5 years recorded to have growth stunting. Sampling was performed using a purposive sampling method leading to the minimal sample size of 80. 18 Data from children aged up to 3 years were excluded from this study.

The secondary data that were reanalyzed were data on eating behavior and caries experience. The eating behavior was modified from the National Health and Nutrition Examination Survey (NHANES) food frequency questionnaire containing 23 questions, including carbohydrates, protein, fat, fast food, fiber, and snacks, coded 1 to 4 for each answer. 19 Eating behavior is the suitability of eating in children that can affect meal times, amount of food intake, food preferences, and food choices involving carbohydrates, protein, fat, fast food, fiber, and snacks. Eating behavior was assessed through a modified WHO food frequency questionnaire. The score is categorized into poor eating behavior if the score is less than population mean and eating behavior is good if the score more than population average.

The DMFT score is used to measure the caries experience of primary teeth by summing up cavities due to caries (decay), teeth extracted due to caries (missing), and teeth filled due to caries (filling). 20 Caries experience is a dental health status that has experienced caries and is assessed using the DMFT scores that were categorized into very low (0–1.1), low (1.2–2.6), moderate (2.7–4.4), high (4.5–6.5), and very high (>6.6).

### Data Analysis

The data were not normally distributed. Data analysis used Spearman Rank correlation test using SPSS v25.0 software with a *p* -value less than 0.05.

### Ethical Aspects

The research has been reviewed and passed the ethical clearance from the Universitas Padjadjaran Research Ethics Commission with document number 172/UN6.KEP/EC/2022.

## Results

The secondary data collected and analyzed for this study are presented in tabular form. The characteristics of the respondents in the secondary data are shown in Table 1 , where the number of boys (51.25) is more than that of girls. Respondents aged 3 years recorded the most compared with other ages (56%). Meanwhile, the response data for the NHANES food questionnaire are collected in Table 2 . It can be seen that most children consume rice, chicken, tempeh/tofu, whole cream milk, vegetable oil, fruit and vegetables, and snacks. The grouping of eating behavior and DMFT score is shown in the same table, namely Table 3 , which shows 63% of respondents have poor eating behavior and also that the majority of respondents have cavities due to caries (decay) 80.35%. Caries is more prevalent in female respondents, 54%, and most caries occur in children aged 3 years (56.47%; Table 4 ). All data are then analyzed and presented in Table 5 .

**Table 1:** Distribution of respondent characteristics by gender and age

Characteristic	*n*	%	
**1. Gender**			
Male	41	51.25	
Female	39	48.75	
**2. Age**			
3 years	45	56	
4 years	35	44	
5 years	0	0	
**Total**	**80**	**100.00**	

**Table 2:** Distribution of modified food frequency questionnaire results

Category	Often				Rarely			
	>1x/day (4)		4–6x/week (3)		<1–3x/week (2)		Never (1)	
	*n*	%	*n*	%	*n*	%	*n*	%
**1. Carbohydrates**								
Rice	80	100	0	0	0	0	0	0
Noodles	0	0	0	0	29	36	51	64
Bread	0	0	1	1	77	96	2	3
**2. Protein**								
Cow	0	0	0	0	29	36	51	64
Goat	0	0	0	0	0	0	80	100
Chicken	39	49	41	51	0	0	0	0
Fish	0	0	1	1	29	36	50	63
Tempe/tofu	75	94	5	6	0	0	0	0
Nuts	0	0	0	0	29	36	51	64
**3. Fat**								
Full cream milk	80	100	0	0	0	0	0	0
Vegetable oil	80	100	0	0	0	0	0	0
Innards	0	0	0	0	0	0	80	100
Cheese	0	0	1	1	29	36	50	63
Butter	0	0	1	1	29	36	50	63
Coconut cream	0	0	0	0	29	36	51	64
**4. Fast food**								
Fast food	0	0	3	4	27	34	50	63
Soft drink	0	0	1	1	28	35	51	64
Fried food	1	1	1	1	28	35	50	63
**5. Fiber**								
Fruit	38	48	42	53	0	0	0	0
Vegetable	36	45	44	55	0	0	0	0
**6. Snack**								
Candy	80	100	0	0	0	0	0	0
Sweet biscuits	80	100	0	0	0	0	0	0
Sweet drink	80	100	0	0	0	0	0	0

**Table 3:** Eating behavior and DMFT score

**No**	**Category**	***n***	**%**
1	Good eating behavior	30	37
2	Poor eating behavior	50	63
**Total**			**80**	**100.00**
**No**	**Category**	**F**	**%**
1	Decay	323	80.35
2	Missing	79	19.65
3	Filling	0	0
**Total**		402	**100.00**

Abbreviation: DMFT, Decayed, Missing, Filled Teeth Index.

**Table 4:** Distribution of DMFT scores based on characteristics

Characteristic	DMFT score	F	%
**1. Gender**			
Male	Decay	185	46
Female	Decay	217	54
**2. Age**			
3 years	Decay	227	56.47
4 years	Decay	175	43.53
5 years	Decay	0	0
Total		402	100,00

Abbreviation: DMFT, Decayed, Missing, Filled Teeth Index.

**Table 5:** The results of statistical analysis of the relationship between eating behavior and caries experience in stunting children in Sukawarna Village, Sukajadi District using Spearman Rank

			Eating behavior	Caries experience
Spearman's rho	Eating behavior	Correlation coefficient	1.000	−0.145
		Sig.(2-tailed)	–	0.0983
		*n*	80	80
	Caries experience	Correlation coefficient	−0.145	1.000
		Sig. (2-tailed)	0.0983	–
		*n*	80	80

## Discussion

Eating behavior is related to eating habits, food selection, and the amount of food eaten. 21 Children of preschool age have the potential to experience psychological development and become toddlers who are independent and can express the emotions they feel. Preschoolers have an active consumption trait. Children can determine what food they want to eat. However, the problem is that children cannot determine which foods are suitable for consumption to meet their nutritional needs. 22

According to Loka, preschoolers can eat with a frequency of three main meals: morning, afternoon, and evening, and are given two small snacks. 23 Based on the questionnaire results, it was found that children always consumed a carbohydrate source in the form of rice more than once a day. This is in line with Febriyani and Mexitalia, children never refuse carbohydrates, and Karyani et al conducted in two kindergartens, found that preschoolers always consume carbohydrates. 22 24 Children always consume protein sources, vegetable, and animal products such as tofu, tempeh, and chicken, while beef, goat, fish, and nuts are rarely consumed. Preschool children need protein to support growth and development. 25 Fat is needed by the body to absorb fat-soluble vitamins, and fat is a crucial source for growth, but if the amount is excessive for a long time will cause excess fat accumulation in the body and the risk of obesity. 25 All children consumed fat in the form of whole cream milk and vegetable oil more than once a day. The questionnaire results found that children rarely consume fast food, soft drinks, and fried foods. This is not in line with Hermina's research at a kindergarten in South Jakarta, which states that there is a tendency for fast food to be consumed by preschool-aged children. 26 The results also show that respondents have a habit of frequently consuming fruits and vegetables every day. This is not in line with the research of Has et al on preschool children in urban areas and Karyani et al conducted in two kindergartens that preschool children rarely consume vegetables and fruit. 22 27 According to Febriyani and Mexitalia, children usually do not like vegetables because there is a bitter taste in them. 24

According to the results of the researcher's analysis, many children's eating behavior is not good because the food consumption of each child is less diverse. According to Scaglioni et al, parents' eating habits and feeding patterns are the most dominant factors in children's eating behavior and food choices. 28 Parents' income levels and mothers' education levels play a role in influencing children's consumption quality. The higher income of the parents will improve eating behavior because of the various foods. A mother's education level can be the basis for making decisions and actions to provide and regulate daily food portions to meet nutritional adequacy. 29

### Caries Experience

From the study results, most children had cavities due to caries (80.35%) compared with teeth extracted due to caries (19.65%), and no teeth were filled. These results are in line with Nabila's research which reported that the most components found in preschool children were decay, missing, and filling. 30

Several factors influenced the high rate of decay: oral hygiene, composition and frequency of food, socioeconomic status, salivary flow rate, and use of fluoride. 10 Saliva contains secretory immunoglobulin A, which protects teeth against caries-causing bacteria. 30 In children, stunting may affect salivary gland atrophy, which causes the decrease of salivary function as a protector; it can make the teeth more susceptible to caries. 30 The absence of filling teeth can be caused by parents' wrong perception regarding replacing primary teeth with permanent ones. 30

Based on Blum's theory, individual oral and dental health is influenced by heredity, environment, behavior, and health services. In Blum's theory, the behavior in maintaining health is influenced by knowledge, attitudes, and actions. 31 This is in line with research conducted by Afrinis et al that there is a significant relationship between a mother's knowledge about dental and oral health and the incidence of caries. The leading cause of caries is an unhealthy lifestyle related to teeth brushing habits. 32 This is in line with the research of Afrinis et al, which said there was a relationship between brushing habits and the incidence of dental caries. 32

Based on the study's results, as stated in Table 5 , caries in women are higher (54%) than in men (46%). This is in line with research conducted by Malaka et al (in Amiqoh) which states that men and women have the same tendency to get caries, but in women, teeth erupt more quickly, causing the teeth to stay longer in the mouth and take longer to have intercourse with caries risk factors. 33 This is not in line with the chi-square analysis conducted by Kali, which states that there is no significant relationship between gender and dental caries. 34

From the study results, most caries occurred at the age of 3 years, which was 56.47%, while at the age of 4 years, it was 43.53%. These results are directly proportional to the chi-square analysis by Ruslan that there is a significant relationship between the child's age and the incidence of caries. This is not in line with research conducted by Pindobilowo (in Ruslan) which states that the younger the child is, the lower the risk of developing caries and Prakash et al (in Ruslan), which states that caries increases with age because of many eruptions of the teeth and consumption of cariogenic foods. 35

### Relationship between Eating Behavior and Caries Experience

Based on the results of the Spearman Rank analysis, the value of *p* = 0.0983 ( *ρ* <0.05). This is because the eating behavior in this study is the current eating behavior of preschool-aged children. Stunting is a state of malnutrition for a long time. Conditions of nutritional status in 1,000 days of life start from when the child is in the womb and continues until at least the first 2 years of postnatal life. These 1,000 days of life can affect the occurrence of stunting because this period is a critical window period for children. 36 At this time, adequate nutritional intake is needed for both mother and child because the growth and development of children occur significantly. Health and maternal conditions are prenatal factors that can cause stunting, while postnatal factors can be caused by infection, environment, and nutrition. 37 Stunting can occur in normal-born babies if their nutritional intake is inadequate. 30 According to Krisnana et al, pregnancy is a prenatal factor related to stunting, while the postnatal factor is exclusive breastfeeding for toddlers. 36 Ramadhandi et al, as stated by Ariati, said there was a significant relationship between food intake, mother's knowledge of complementary foods and the nutritional status of stunting toddlers. This is in line with Ariati, who said that maternal nutrition status during pregnancy, history of exclusive breastfeeding, and protein intake cause stunting. 38

Caries is a multifactorial disease. 39 The hosts that are closely related to the occurrence of caries are tooth structure and saliva. In children, stunting is a host factor related to dental structural abnormalities, enamel hypoplasia, and delayed tooth eruption, so children are more susceptible to demineralization and caries. 30 40 Deficiency of vitamins D and A and protein-energy malnutrition can cause enamel hypoplasia and salivary gland atrophy. 41 Salivary gland atrophy causes decreased saliva production and interfere with its function as a buffer, cleanser, and antibacterial, which will increase the risk of caries. 42 This is in line with research by Achmad et al that there is a relationship between stunting in children with oral health conditions, including the incidence of caries, delayed eruption of permanent teeth, and affecting the rate of salivary flow. Stunting in children and dental health conditions are interconnected. 10

Aviva et al, in their research, stated that stunting children had a higher caries rate in primary teeth than normal children. 43 In Nabila's study, stunting children had an average caries experience score of two times higher than the control group. 30 Rahman and Adhani in their research stated that the mean DMFT scores in stunting children are almost three times higher than in normal children. 42 Stunting children have a 3.4 times higher chance of experiencing caries than normal nutritional children. The results of the study contained in Table 2 show that respondents always consume sweet snacks and sweet drinks with a frequency of more than once a day, and this is following the research of Karyani et al which states that children generally like sweet foods. 22 Dental caries in preschool children is because children have a habit of consuming cariogenic foods, and if left for a long time, will cause caries. 32

Cariogenic foods contain fermented carbohydrates that can cause plaque pH to drop to 5.5 or less and can encourage caries. 44 The chi-squared test analysis conducted by Ismail said there was a significant relationship between the consumption of cariogenic foods and the incidence of dental caries. 34 These results are in line with Afrinis et al who stated that there is a significant relationship between sweet foods and the occurrence of dental caries. 32 From the study results, the effect of eating behavior on the caries experience in stunting children is 2.1%, or it can be concluded that they have a nonsignificant relationship. At the same time, the remaining 97.9% is influenced by other factors, for example, host factors, economic status, fluoride use, mother's efforts to maintain the child's dental health, and how to brush teeth correctly by mother.

## Conclusion

Consumption of cariogenic foods influences the caries experience. Although, statistically, eating behavior has no relationship with caries experience, overall, eating behavior has a relationship with caries experience in stunting children. In this study, there were no respondents aged 5 years. The data used is secondary data from the modified WHO food frequency questionnaire, so that researchers cannot know the daily consumption portion of the type of food consumed. Further research needs to be conducted with a broader scope regarding the eating behavior of stunting children of preschool age, for example, eating behavior and tooth brushing behavior with caries experience in stunting children.
